# Case of Oedema Fugax

**Published:** 1801-08

**Authors:** J. Yelloly

**Affiliations:** Bartlett's Buildings, Holborn


### *Case of Oedema Fugax.* Communicated by Dr. Yelloly, Phyfician to the General Difpenfary.

---

To the Editors of the Medical and Physical Journal.

Gentlemen,

In your Journal of laft month, particulars are mentioned, by Dr. Crichton, of a difeafe which has not hitherto been defcribed by practical or nofological writers, to which he gives the name of Oedema Fugax. A cafe of a fimilar kind occurred to a medical friend in the city a fhort time ago; and as I was induced, in confequence of that related by Dr. Crichton, to make more particular enquiries concerning it, both from my friend and the patient himfelf, the refult may not, perhaps, haps, be unworthy of infertion in the Medical Journal, as an additional inftance of a rare and obfcure difeafe.

Mr. C. of Gower Street, a married man, under thirty, went to bed on the 12th of June laft in perfect health. He awoke at three or four in the morning, and was furprifed to find himfelf affected with a confiderable fwelling in the fcrotum, unattended with pain. This fingular and unexpected occurrence made him uneafy, and prevented him from fleeping: Soon afterwards he felt the penis begin to fwell, and the fcrotum to decreafe; and on rifing in the morning, the penis was fwelled to an enormous fize, fo as to alarm him very much, while the fwelling of the fcrotum could fcarcely be obferved. My friend faw him in the courfe of the morning, at which time the fwelling feemed to be at its height. It was free from pain, heat, or rednefs, with no perceptible pitting on preffure, or fenfe of fullnefs: The fkin was tranfparent, and particularly diftended at the frænum, which formed the end of the fwelling: The prepuce could not be retracted. In other refpects he was in perfect health. A purgative medicine and faturnine lotion were ordered, and in the evening the penis was confiderably reduced in fize, but a flight fwelling was obferved over the right eye. This was very perceptible in the morning, but had not increafed, and the penis was ftill more diminifhed. In the courfe of the day the fwelling of the eye difappeared, and in the following morning the penis was nearly in its natural ftate.

About a week from that time, after riding fome miles on horfeback, he obferved in the evening a fwelling, attended with flight pain and rednefs, in the upper and fore part of the left arm, not completely encircling it. In the following morning the left arm was unchanged, but the right was affected down to the hand, with confiderable fwelling and fenfe of fullnefs. This was next day much better, but the fwelling near the fhoulder in the left arm continued; and another appeared below the elbow, which fpread to, and affected the hand, and was attended with flight pain, itching, and fenfe of fullnefs, particularly on moving the arm. The elevation was uniform through the whole of the affected part, and in none of them was there any perceptible pitting on preffure. The tumour in the left fore arm was fomewhat variegated in colour, and its increafe was fo rapid as to be clearly difcernible on viewing it, after withdrawing the eye but for a very fhort time. This laft fwelling did not continue longer than thofe which preceded it, but before it entirely difappeared, he was affected with another under his chin, which was very large, hard, colourlefs, and without pain. It gradually advanced to the under lip, which it diſtorted, and, as he ſays, diſtended like a bladder, ſo as to alarm him much. In the courſe of the night, on awaking, he found that the lower lip was much fallen, and the upper as much ſwelled as the lower had been: By the evening of the following day, little of either of thoſe remained.

During the time of his being affected in the way which I have deſcribed, he occaſionally had ſlight and tranſient ſwellings of the knee, ancle, and foot; and when I ſaw him, which was on July 6, he had ſome degree of fullneſs in the inſide of the fingers, which had been more conſiderable the day before. He had the advice of two very eminent practitioners ſoon after he was firſt attacked, and, at their deſire, took ſaline medicines, and afterwards tonics, without much apparent advantage. His general health through the whole complaint remained perfectly unaffected.

It may appear, perhaps, unneceſſary to have gone ſo much into the detail of this caſe; I thought it better, however, to give it in the preciſe and diſtinct mode in which it was related to me by my friend and the patient, (both of whom are men of ſenſe and obſervation) than merely ſtate it to you in general terms.

It ſeems to have been analogous to that of Dr. Crichton's in every particular but the want of general indiſpoſition preceding and accompanying the appearance of the ſwellings.

